# Paradoxical positive association of serum adiponectin with all-cause mortality based on body composition in Japanese haemodialysis patients

**DOI:** 10.1038/s41598-018-33011-y

**Published:** 2018-10-02

**Authors:** Yuri Machiba, Masaaki Inaba, Katsuhito Mori, Masafumi Kurajoh, Kozo Nishide, Kyoko Norimine, Tomoyuki Yamakawa, Shigeichi Shoji, Senji Okuno

**Affiliations:** 1 Department of Metabolism, Endocrinology and Molecular Medicine, Osaka City University Graduate School of Medicine, Osaka, Japan; 2 Department of Nephrology, Osaka City University Graduate School of Medicine, Osaka, Japan; 3 Kidney Center, Shirasagi Hospital, Osaka, Japan

## Abstract

We have previously reported a paradoxical association of serum adiponectin with aortic calcification in haemodialysis patients. Because serum adiponectin is a nutritional marker, we examined the association between serum adiponectin and all-cause mortality based on body composition in haemodialysis patients. The trunk and total body fat were determined. The patients were divided into two groups based on serum adiponectin levels. In Kaplan–Meier analysis, the higher adiponectin group showed higher mortality than the lower adiponectin group. Serum adiponectin showed an inverse correlation with the percentage of truncal fat, suggesting serum adiponectin as an inverse marker for adiposity in haemodialysis patients. However, even after adjustment for other factors, multivariate Cox proportional hazards analysis identified higher serum adiponectin as an independent factor positively associated with higher mortality in haemodialysis patients. This association held true even when the total fat mass was replaced with the percentage of truncal fat, and when total fat mass and percentage of truncal fat were simultaneously included. Thus, we found a paradoxical association of higher serum adiponectin with higher all-cause mortality in Japanese haemodialysis patients, independent of adiposity.

## Introduction

We have previously reported the reversal of an inverse association between serum adiponectin and abdominal aortic calcification in the general population to a positive association in haemodialysis patients^1^, although accumulated evidence indicates that higher serum adiponectin might predict lower cardiovascular disease (CVD) risk in the general population and in diabetes mellitus (DM) patients^2,3^. Because it is well known that better nutritional status predicts lower mortality in haemodialysis patients^4^, it is possible that increase in serum adiponectin resulting from decreased visceral adiposity in malnourished patients might be influenced by malnutrition-associated exacerbation of mortality in haemodialysis patients^5^. We previously reported that increase in either fat mass or lean mass, determined using dual-energy X-ray absorptiometry (DXA), might provide a reliable prediction of better survival in haemodialysis patients^6^. Our previous findings of a significant and positive association of handgrip strength and serum creatinine level with better survival in DM or non-DM haemodialysis patients^7,8^ are supportive of this notion. Because a series of previous reports have shown the development of adiponectin resistance following receptor activation in chronic kidney disease (CKD) patients^9^, it is important to elucidate the mechanism(s) that might contribute to the reversal of the inverse association observed in the general population mentioned above to a positive one in haemodialysis patients.

This background prompted us to investigate the association between serum adiponectin and all-cause mortality in haemodialysis patients based on body composition such as total fat mass and percentage of truncal fat mass.

## Results

### Clinical and biochemical profiles of the participated haemodialysis patients

The baseline characteristics of the 113 haemodialysis patients are shown in Table 1. The mean age (±standard deviation: SD) of all participants was 61.6 ± 10.8 years and the duration of haemodialysis was 6.5 ± 3.0 years. Body weight, body mass index (BMI), fat mass and percentage of truncal fat mass values were 59.3 ± 9.4 kg, 21.9 ± 3.0 kg/m^2^, 12.7 ± 5.8 kg and 46.3% ± 8.3%, respectively. The median serum adiponectin level was 17.6 (interquartile range: IQR, 11.7–24.5) μg/mL, which is approximately 3-fold higher than the reported value of 5.4 ± 2.3 µg/mL in non-CKD male subjects^10^.

**Table 1:** Clinical and biochemical characteristics.

	All (n = 113)	Lower adiponectin (n = 57)	Higher adiponectin (n = 56)	p value
Age (years)	61.6 ± 10.8	61.8 ± 10.6	61.4 ± 11.0	0.8315
Duration of HD (years)	6.5 ± 3.0	6.1 ± 2.9	6.9 ± 3.0	0.1683
Diabetes mellitus (yes/no)	50/63	26/31	24/32	0.9146
Body weight (kg)	59.3 ± 9.4	60.9 ± 10.3	57.5 ± 8.2	0.0686
Body mass index (kg/m^2^)	21.9 ± 3.0	22.4 ± 3.3	21.3 ± 2.6	0.0585
Fat mass (kg)	12.7 ± 5.8	14.3 ± 5.9	11.1 ± 5.1	0.0026
Percent truncal fat mass (%)	46.3 ± 8.3	49.2 ± 8.1	43.4 ± 7.5	0.0001
Albumin (g/dL)	3.8 ± 0.3	3.8 ± 0.2	3.8 ± 0.3	0.3248
hs-CRP (μg/dL)	119 (46–470)	107 (40–396)	122 (62–691)	0.9146
Adiponectin (μg/mL)	17.6 (11.7–24.5)	11.7 (8.0–15.0)	24.7 (20.9–32.3)	<0.0001

Data are expressed in mean ± SD, or median (IQR).

Differences in mean and median values were examined by Student’s t-test and the Mann–Whitney U test, respectively.

### Comparison of clinical variables between haemodialysis patients with higher and lower serum adiponectin

We next examined the relationship of serum adiponectin with all-cause mortality in haemodialysis patients. The patients were divided into two groups based on their median (17.6 µg/mL) serum adiponectin level; those with lower serum adiponectin (<17.6 µg/mL) and those with higher serum adiponectin (≧17.6 µg/mL), and the clinical variables in these groups were compared (Table 1). Among the various clinical variables examined, the BMI values of the patients with lower and higher serum adiponectin levels were 22.4 ± 3.3 and 21.3 ± 2.6 kg/m^2^, respectively, with no significant difference. The total fat mass and percentage of truncal fat mass were significantly greater in the lower adiponectin group than in the higher adiponectin group. Serum adiponectin was significantly correlated in a negative manner with the total fat mass (ρ = −0.401, p < 0.0001), and percentage of truncal fat (ρ = −0.456, p < 0.0001) in the patients (data not shown), in agreement with a similar significant correlation reported previously in the general population^11^, suggesting serum adiponectin as a clinically relevant marker of adiposity even in haemodialysis patients.

### Kaplan–Meier analysis

**Figure 1:**
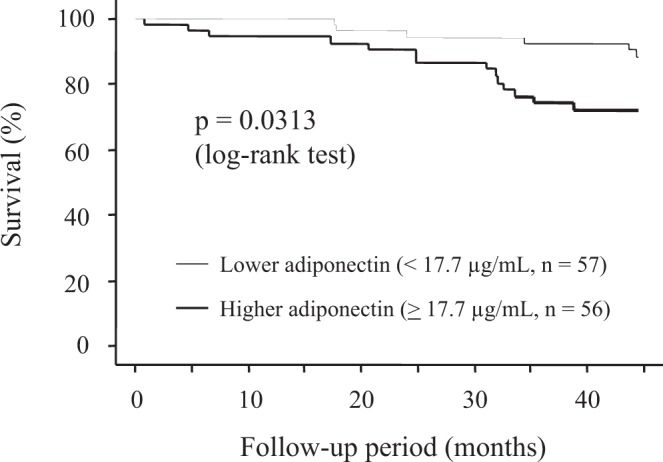
Kaplan–Meier analyses to examine the association between serum adiponectin and all-cause mortality. Patients with higher serum adiponectin (≧17.7 μg/mL) experienced a significantly higher rate of all-cause death than those with lower adiponectin (<17.7 μg/mL) (p = 0.0313 in log-rank test).

During the mean follow-up period of 38 months, 20 patients (18%) died, leaving 93 (82%) surviving patients by the end of the study period. The patients who died comprised 14 (25%) of the 56 patients with higher adiponectin and six (11%) of the 57 patients with lower adiponectin. The Kaplan–Meier analyses performed to examine the association between serum adiponectin and all-cause mortality (Fig. 1) demonstrated that patients with higher adiponectin experienced a significantly higher rate of all-cause mortality than those with lower adiponectin (p < 0.0313 in log-rank test).

### Multivariate analysis with Cox proportional hazards models

Multivariate Cox proportional hazards analysis was performed to confirm serum adiponectin as an independent factor associating positively with all-cause mortality and to examine whether this positive association might be influenced by markers of nutritional status such as serum albumin, body fat and/or truncal fat (Table 2). Model 1, which included log-adiponectin and total fat mass in addition to age, haemodialysis duration, presence/absence of DM, serum albumin and log-high-sensitivity C-reactive protein (hsCRP) as independent variables, showed log-adiponectin as a significant and independent factor associating positively with all-cause mortality, in addition to total fat mass, presence/absence of DM and log-hsCRP [odds ratio (OR), 13.542; 95% confidence intervals (CIs), 1.289–142.242; p = 0.0299]. Model 2, which included the percentage of truncal fat instead of the total fat mass, demonstrated that log-adiponectin, in addition to percentage of truncal fat, presence/absence of DM and log-hsCRP, emerged again as a significant and positive factor associating with all-cause mortality (OR, 12.064; 95% CI, 1.151–126.438; p = 0.0377). When the total fat mass and percentage of truncal fat were simultaneously included as independent variables in Model 3, both failed to associate with all-cause mortality while log-adiponectin retained its significant and positive association with all-cause mortality (OR, 11.488; 95% CI, 1.091–121.015; p = 0.0421).

**Table 2:** Multivariate Cox proportional hazards analyses to elucidate serum adiponectin as an independent factor to associate positively with all-cause mortality in haemodialysis patients.

	Model 1			Model 2			Model 3		
	HR	95% CI	p	HR	95% CI	p	HR	95% CI	p
Age (yr)	1.044	0.979–1.113	0.1892	1.041	0.979–1.106	0.2003	1.014	0.997–1.108	0.2160
HD duration (yr)	0.981	0.840–1.145	0.8053	0.967	0.822–1.137	0.6835	0.996	0.823–1.135	0.6760
Diabetes (yes)	4.321	1.413–13.210	0.0103	4.987	1.619–15.360	0.0051	4.885	1.584–15.062	0.0058
Albumin (g/dL)	2.450	0.322–18.647	0.3868	2.380	0.322–17.601	0.3956	2.484	0.331–18.622	0.3758
log [hsCRP (μg/dL)]	5.555	2.309–13.364	0.0001	7.037	2.714–18.245	<0.0001	6.995	2.684–18.229	<0.0001
Log [adiponectin (μg/mL)]	13.542	1.289–142.242	0.0299	12.064	1.151–126.438	0.0377	11.488	1.091–121.015	0.0421
Total fat mass (kg)	0.874	0.775–0.986	0.0291	—	—	—	0.957	0.818–1.120	0.5844
Truncal fat (%)	—	—	—	0.899	0.832–0.972	0.0072	0.921	0.823–1.032	0.1576

## Discussion

The present study demonstrated that higher serum adiponectin, a factor known to be protective against atherosclerosis in the general population^12,13^, was paradoxically associated with higher all-cause mortality in Japanese haemodialysis patients (Fig. 1). Because these findings confirmed our previous finding that serum adiponectin correlated inversely with total fat mass and percentage of truncal fat in Japanese male haemodialysis patients^14^ and in the general population, they suggest the possibility that improved nutritional status reflected in higher total fat mass and percentage of truncal fat may induce suppression of serum adiponectin while improving clinical outcomes in such patients. Therefore, it is important to examine whether such a reversal of the inverse association between serum adiponectin and mortality in these patients might be confounded by their adiposity characteristics. The present study demonstrated that higher adiponectin level represents a clinically relevant marker for poor survival in haemodialysis patients, independently of total fat mass and/or percentage of truncal fat mass in addition to age, haemodialysis duration, presence/absence of DM, serum albumin and log-hsCRP. This suggests that the paradoxical association of higher serum adiponectin with higher all-cause mortality in haemodialysis patients cannot be explained by the increase in serum adiponectin resulting from reduction in fat mass. Previous reports have shown that higher adiponectin is associated with higher death risk in haemodialysis patients independent of body composition and lipids^15^. However, because such studies enrolled patients who were relatively obese compared to ours (median BMI was 29.0 kg/m^2^) and their body composition was measured using body anthropometry, the influence of the nutritional status and increased body fat on the association between serum adiponectin and mortality could not be completely excluded. In contrast, because the present study restricted the participated patients to relatively non-obese patients and applied DXA to precisely measure total fat and percentage of truncal fat, the effect of the adiposity status might have been avoided better. Further, BMI did not differ significantly between patients with lower serum adiponectin and those with higher adiponectin (Table 1), although the total fat mass and percentage of truncal fat were significantly higher in those with lower adiponectin. Thus, the present study raises the possibility that higher serum adiponectin might directly contribute to a survival disadvantage in relatively non-obese haemodialysis patients. Although adiponectin exhibits significant anti-diabetic, anti-inflammatory and anti-atherogenic properties^2,3^, CKD patients, who exhibit a serum adiponectin level approximately 3-fold higher than the reported value of 5.4 ± 2.3 µg/mL in non-CKD subjects^1^, are at higher risk for all-cause mortality. Of significant interest, the present study demonstrated that even in relatively non-obese haemodialysis patients, those with higher serum adiponectin are at significant risk for higher all-cause mortality, independently of total fat, truncal fat, or serum albumin (Table 2). We had reported previously that higher serum adiponectin was associated with reduced bone mineral density^14^ and higher prevalence of abdominal aortic calcification in haemodialysis patients^1^, suggesting a paradoxical association of adiponectin with all-cause mortality, atherosclerosis and osteoporosis in haemodialysis patients, even after adjustment for their body fat.

Several mechanisms have been hypothesised to explain the reversed association of serum adiponectin with mortality in CKD patients. First, accumulated evidence indicates the development of adiponectin resistance in CKD patients as evidenced from *in vivo* or *in vitro* data. It was previously reported that acetyl-CoA carboxylase phosphorylation and carnitine palmitoyl transferase 1 levels, which are downstream effectors of adiponectin, were lower although the tissue levels of the adiponectin receptor 1 were higher in CKD patients, indicating the development of adiponectin resistance in CKD after receptor activation following the phosphorylation of 5′ adenosine monophosphate-activated protein kinase^9^. Alternatively, the accumulation of biologically inactive adiponectin in uremic serum might be postulated. However, the previous finding that increase in serum adiponectin is explained by its increased production in fat tissue but not by its decreased renal clearance negated this possibility^16^.

The strength of the present study was that the participated subjects were male, and that BMI did not differ between patients with higher serum adiponectin and those with lower serum adiponectin, possibly minimising the influence of gender and truncal and total fat levels on the association between serum adiponectin and all-cause mortality. If large differences in fat mass are present in participated patients, the influence of total fat and/or truncal fat cannot be completely negated even after adjusting for them. Thus, the present study has reliably demonstrated the paradoxical association of higher serum adiponectin with higher all-cause mortality in haemodialysis patients independent of nutritional status including total fat mass, percentage of truncal fat, BMI and serum albumin. Further, the study was performed in a single institution, whereby effects of any differences in management and treatment policy on nutritional status and mortality of the participated patients could be minimised.

There are several limitations in the present study. First, because of its observational nature, the associations do not necessarily indicate causality. Therefore, although the results of this study are of clinical interest, randomised controlled trials are needed to prove its hypothesis. Second, all patients were Japanese men and the sample size was relatively small, which may limit extrapolation to other ethnic groups. Third, recent studies have reported several adiponectin isoforms with different metabolic activities; we did not address this in the present study. Fourth, there was no information about medications and smoking status that could modulate adiponectin levels. Lastly, we calculated the mortality risk based on single-point measurements of serum adiponectin at the start of the study, not averaged values during the follow-up period. Therefore, we could only demonstrate a remote effect on mortality during follow-up. Time-averaged analysis based on sequential measurements during the follow-up may provide more direct evidence of association between serum adiponectin and clinical outcomes.

In conclusion, we found that serum adiponectin was paradoxically associated in a positive manner with all-cause mortality in Japanese male patients on haemodialysis, independent of adiposity.

## Subjects and Methods

### Patients

The subjects were Japanese patients maintained on haemodialysis (n = 113) and treated at the Shirasagi Hospital. The participated patients were restricted to males. Informed consent was obtained from each patient and the study protocol was approved by the Ethics Committee of the Shirasagi Hospital^14^ and conducted in accordance with the principles of the Declaration of Helsinki. Patients who had been treated with haemodialysis for less than one year or for more than 21 years were excluded from the study based on a previous report^17^. Those who had acute illness, infection, or malignancy were also excluded. The diagnosis of DM was based on a history of DM or meeting the criteria for DM as defined in the Report of the Expert Committee on the Diagnosis and Classification of Diabetes Mellitus^18^.

### Sample collection

Patients underwent haemodialysis sessions three times weekly on Monday, Wednesday and Friday. Blood sampling was performed during the Monday session, exactly 68 h after the previous (i.e., Friday) session^1^. A blood sample was withdrawn from the arteriovenous fistula immediately before the start of the haemodialysis. The sample was placed at room temperature for 1 h and then centrifuged at 1,000 × *g* for 10 min. The resultant serum was stored in aliquots at −20 °C until assayed. The frozen samples were thawed and assayed immediately. Serum hs-CRP was measured using a nephelometric assay (SRL Inc., Tokyo Japan). Serum adiponectin levels were measured using a human adiponectin ELISA kit (Otsuka Pharmaceuticals Co., Tokyo, Japan)^1,19^.

### Body composition measurements

Fat levels in the total body, trunk, arms and legs were measured using DXA (QDR-4500; Hologic, Waltham, MA, USA) 21–24 h after completion of a dialysis session^20^, percentage body fat values were calculated using the built-in software of the DXA instrument, as previously reported^21–23^. The reproducibility of measurements expressed by coefficient value for fat mass using DXA was less than 2%, as previously reported^22^. BMI was defined as the patients’ weight in kilograms divided by the square of their height in metres.

### Statistical analysis

Data were expressed as mean ± SD or median (IQR). The normality of the variables was assessed. Differences in the mean and median values were evaluated using the Student’s *t*-test and the Mann–Whitney *U* test, respectively. Survival curves were constructed using the Kaplan–Meier method and evaluated with the log-rank test. Prognostic variables for survival were examined using multivariate Cox proportional hazards regression models and independent predictors of death were determined using multivariate Cox analyses. Because the distributions of hs-CRP and adiponectin were skewed, they were log-transformed to obtain normal distributions. The OR with 95% CIs was calculated. P < 0.05 was considered as the threshold for significance. All analyses were performed using StatView 5 for Windows (SAS Institute Inc., Cary, NC, USA).
